# The Coming Primary Care Revolution

**DOI:** 10.1007/s11606-016-3944-3

**Published:** 2017-02-27

**Authors:** Andrew L. Ellner, Russell S. Phillips

**Affiliations:** 1 Center for Primary Care, Harvard Medical School, 635 Huntington Avenue, Boston, MA 02115 USA; 2 Division of Global Health Equity, Brigham and Women’s Hospital, Boston, MA USA; 3 Division of General Medicine and Primary Care, Beth Israel Deaconess Medical Center, Boston, MA USA

**Keywords:** primary care, healthcare systems, value-based care, care delivery innovation, health workforce

## Abstract

The United States has the most expensive, technologically advanced, and sub-specialized healthcare system in the world, yet it has worse population health status than any other high-income country. Rising healthcare costs, high rates of waste, the continued trend towards chronic non-communicable disease, and the growth of new market entrants that compete with primary care services have set the stage for fundamental change in all of healthcare, driven by a revolution in primary care. We believe that the coming primary care revolution ought to be guided by the following design principles: 1) Payment must adequately support primary care and reward value, including non-visit-based care. 2) Relationships will serve as the bedrock of value in primary care, and will increasingly be fostered by teams, improved clinical operations, and technology, with patients and non-physicians assuming an ever-increasing role in most aspects of healthcare. 3) Generalist physicians will increasingly focus on high-acuity and high-complexity presentations, and primary care teams will increasingly manage conditions that specialists managed in the past. 4) Primary care will refocus on whole-person care, and address health behaviors as well as vision, hearing, dental, and social services. Design based on these principles should lead to higher-value healthcare, but will require new approaches to workforce training.

## INTRODUCTION

Primary care has been described as “integrated, accessible health care services by clinicians who are accountable for addressing a large majority of personal health care needs, developing a sustained partnership with patients, and practicing in the context of family and community.”^1^ This and other seminal definitions of primary care do not specify a type of clinician, but rather refer to the set of essential functions which primary care serves within healthcare systems—namely, access, continuity, comprehensiveness, and coordination.^1^^–^ 3

Legislative reform, technological evolution, shifting public expectations, and pressure for cost discipline have set the stage for accelerating change for healthcare systems. Primary care requires a compelling vision and profound changes to thrive. We argue that primary care serves critical functions that will be as vital in the future as they have been in the past, and that these functions may be more optimally achieved through different configurations of people and technology, guided by four principles.

## WHY DO HEALTHCARE SYSTEMS NEED PRIMARY CARE?

The major frameworks^4^^–^ 6 that conceptualize healthcare systems converge on the idea that healthcare systems should produce better health outcomes and patient experience at a sustainable cost. The World Health Organization’s framework additionally embraces equity as a primary aim of health systems by including the goals of financial and social risk protection and the fair distribution of health outcomes across populations. While all of the world’s healthcare systems struggle to achieve these aims, and experience different tradeoffs between healthcare cost, quality, access, and equity, the United States has the most expensive, technologically advanced, and sub-specialized healthcare, with worse population health outcomes and measures of equity^7^ than any other high-income country.^8^

Acknowledging the predominant role of factors outside healthcare in determining the health of individuals and populations,^2^^–^ 9 there is also convincing evidence that geographic areas with a higher concentration of primary care providers demonstrate better health outcomes, better healthcare quality, lower total medical expenditures, and more equitable health outcomes.^3^ The primary care functions of access, continuity, comprehensiveness, and coordination are each associated with improved care processes and outcomes (Table 1). As non-physician health workers can serve many aspects of primary care functions, including diagnosis and management, with equal or greater reliability and at lower cost, working as a team may create the highest value. Consistent evidence suggests that nurse practitioners and physician assistants perform many of the clinical roles in primary care as reliably as physicians, including the care of relatively complex patients.^10^^–^ 12 Care managers (typically nurses), community health workers, and patient navigators can prevent emergency room visits and hospitalizations as they work with primary care teams to coordinate and optimize health service utilization^13^; they may also help ensure patient adherence to medical regimens and reduce disparities in care by helping patients to overcome barriers, such as lack of transportation, low English literacy, or difficulty accessing social services.^14^

**Table 1:** Selected Evidence That Primary Care Functions Are Associated with Improved Outcomes

Function	Outcome evidence
Access (first contact care for new health needs)	Reduces unnecessary hospitalizations,^59^ improves health, reduces non-urgent ED and specialist visits,^60^ improves rates of immunization, preventive services, and counseling^61^
Continuity (long-term person-focused care)	Improves chronic disease management and use of preventive services,^62^ reduces ED use and hospitalizations,^63^^,^ 64 improves quality of care and reduces cost^65^
Comprehensiveness (care for most health needs)	Reduces cost, 66 ^,^ 67 subspecialty visits^66^ and ﻿hospitalizations,^67^^,^ 68 improves rates of immunizations, preventive screening and counseling^61^
Coordination of care (when required outside the practice)	Reduces costs and hospitalizations,^67^^,^ 69 improves specialty referrals 70

*ED* emergency department

New models of care delivery, supported by legislative reform, reinforce the potential for primary care-based approaches to improve value. The patient-centered medical home (PCMH) is a construct for team-based primary care oriented towards improving the health of a panel of patients. Although outcomes vary, PCMH demonstration projects have shown promising reductions in costs and improvements in quality, attributable in part to effective teamwork.^15^ Early evidence regarding accountable care organizations indicates that those built around primary care, and therefore better positioned to negotiate a reduction in the costs of specialty and hospital services, fare better than those built around hospitals.^16^

## WHY DO WE NEED A PRIMARY CARE REVOLUTION?

In the U.S., prior to the quite recent preliminary changes in the direction of paying for value, healthcare financing has focused almost exclusively on payment for transactional, procedural care. Some have argued that the relative value unit (RVU) schedule for primary care visits and other cognitive activities undervalues the role of primary care and the skills and experience required of primary care practitioners and teams.^17^^–^ 19 Marginal finances and other external constraints, as well as internal limitations of primary care practices, result in challenging work–life balance, high rates of physician and staff burnout, workforce shortages, poor quality of care, and lower salaries and prestige compared to other specialties. Not surprisingly, given the low revenue streams and high expectations for unreimbursed labor, many primary care practices struggle to maintain financial sustainability.^20^ Practices that are succeeding financially are often doing so as a result of investment by a health system that values the patients cared for within the practice, but most of these systems are still paying for primary care services using the Medicare RVU schedule, leaving few options for practices to add high-value services that are not currently reimbursed. Some experts anticipate a sizable primary care workforce shortage,^21^ while others suggest that this shortfall could be moderated by changes in staffing models to accommodate greater provision of primary care by advance practice practitioners.^22^ However, nurse practitioner and physician assistant trainees face the same disincentive to choose primary care as do medical students.^23^

The forces creating pressure for change in healthcare systems are likely to increase. Most importantly, healthcare expenditures will continue to grow, and may again outpace overall economic growth, due to reinvigorated biomedical technological innovation, an increasing number of Americans receiving health insurance, the epidemic of chronic disease, which accounts for 86% of overall expenditure,^24^ and the growing proportion of Americans over the age of 65. There is also evidence of rising consumerism, whereby Americans increasingly expect a level of service from healthcare commensurate with other products and services and, at least for basic, acute care, value convenience over the reputation and expertise of providers.^25^^,^ 26

In the crucible created by forces for change, along with the unparalleled opportunity provided by the free enterprise system, it is not surprising that there are growing threats to traditional healthcare provision by new market entrants. Established market incumbents (such as pharmacy chains) as well as startups see the rampant waste of up to 40%,^27^ healthcare’s unparalleled lack of growth in labor productivity,^28^ the poor consumer-friendliness of incumbent healthcare providers, and the potential applicability of advances such as artificial intelligence as a business opportunity. The emergence of urgent care in retail pharmacies and standalone chains is an early example of how new business lines are beginning to infringe on aspects of care that have traditionally been the purview of primary care practices.^26^ Several telemedicine companies offering urgent transactional care over secure virtual platforms have also entered the market. While uptake of these telemedicine services has been relatively limited, they reflect the reality that, as long as traditional primary care practices fail to adequately meet patients’ expectations and needs, new market entrants will attempt to fill this void.

Some have suggested a future for healthcare wherein the majority of highly empowered consumers will bypass primary care altogether and choose among a massive array of highly specialized services and technologies for illness care and wellness support.^29^ The preponderance of evidence suggests, however, that in the absence of the critical primary care functions, healthcare systems employing this approach will experience more fragmentation of care, duplication, and waste, as well as more inequitable health outcomes. Instead, we believe that strong longitudinal relationships will continue to be a major source of value in healthcare that directly contributes to technical excellence in the prevention, diagnosis, and treatment of disease; supports healthy lifestyle choices and adherence to beneficial treatment regimens, particularly for those whose agency is most constrained by structural inequality and other life circumstances; and holds significant independent value in patients’ experience of care.^30^

## WHAT PRINCIPLES SHOULD GUIDE THE PRIMARY CARE REVOLUTION?

The primary care revolution must respond to these forces, facilitated by changes in payment, practice redesign, and innovative uses of technology.^31^ Primary care must assert itself as the only viable solution to the interrelated problems of rising costs, renewed biomedical technological innovation in the direction of more personalization, public demand for convenience, and widespread waste. The essential functions of primary care will be just as relevant to the future of healthcare as they have been up to now. Thus, the task at hand is to optimize for these functions, in addition to technical excellence in the prevention, diagnosis, and treatment of disease. Doing so in a way that enables financially sustainable care at the massive scale needed to adequately serve all U.S. citizens will require an unsentimental reexamination of how the competencies, actions, information, and power in primary care and the rest of healthcare are distributed among people and technology. We concur with others^32^ that the next few decades will witness a significant transfer of power, knowledge, and activity from the most specialized providers to patients, with primary care serving as the key fulcrum in the transfer. This is not to say that there will be no specialists in the future, but rather that they will increasingly be leveraged to assist with the most complex diagnostic and management challenges, while primary care teams and patients are empowered to manage routine cases.

To inform healthcare stakeholders, we offer four principles to guide the primary care revolution. These principles are derived from our own experience as primary care physicians and leaders, synthesis and analysis of existing evidence (including what already works well in service industries outside of healthcare), and knowledge derived from an in-progress program of mixed-methods research studying high-functioning primary care systems.^33^ (See Table 2 for an illustration of how the principles would manifest in the care of a patient.)

**Table 2:** Illustrating the Four Principles in the Care of a Patient

Patient history: Ms. W • 35-year-old single mother of two • Works nights as service professional • History of abusive relationships; current boss is emotionally abusive • Smokes 10 cigarettes daily; binge-drinks on weekends • Family history of colon cancer in two first-degree relatives including her father at age 42, lupus, diabetes, and depression • Intermittent mild–moderate depression and poorly controlled diabetes and hypertension • Frequently misses medical appointments					
Health state and example	Principle 1: Payment Reform towards Capitation	Principle 2: Team- and technology-supported relationships	Principle 3: Integration with specialists	Principle 4: Whole-person care	Outcome and impact
Baseline health: • Ms. W’s daughter gets on her case about smoking, and she decides she would like to try and quit	• She shares her interest in quitting smoking during her twice-yearly email check-in by her health coach	• Her health coach forwards an evidence-based decision support and interactive tool on different pharmacotherapy options for smoking cessation • Her NP prescribes nicotine patches, which Ms. W has chosen • Her health coach also identifies mild depression based on Ms. W’s answers to PHQ-2 and PHQ-9	• Her physician is focused on complex presentations by other patients, but based on a short conversation with the health coach, suggests a referral for CBT	• An LICSW reaches out to her, and they schedule time for an initial telephonic CBT session	• Ms. W fails to quit smoking this time • She does experience improvement in her depression, however, as indicated by follow-up PHQ-9s • Three years later, after one other unsuccessful quit attempt, she finally succeeds
Acute, routine care: • Ms. W develops dysuria and suprapubic discomfort • “Feels like when she had a UTI” • No fevers, back pain, nausea or vomiting	• Ms. W emails the triage line for her practice • As there are no red flags, her care is managed virtually	• An interactive technology takes the basic history • Her NP calls Ms. W, confirms the history, prescribes antibiotic	• Her physician is focused on complex presentations by other patients	• Ms. W’s health coach calls her in 5 days to make sure she is feeling better • He reminds her that she is due for her colonoscopy and forwards a scheduling link	• Ms. W’s UTI symptoms completely resolve within 36 h • She does not have to use any of her sick time at work • After four more monthly automated email reminders, she does schedule and go for a colonoscopy, which is normal
Chronic disease: • Ms. W develops her third yeast infection in a year • She has not had a hemoglobin A1C test in 9 months	• Ms. W’s health coach is notified that she is overdue for her hemoglobin A1C test and contacts Ms. W to urge her to get the test • Her hemoglobin A1C comes back at 8.8	• Ms. W’s health coach checks in with Ms. W about her diet and schedules a virtual check-in with her NP • Her NP reviews her medications and learns that she is taking her sulfonylurea at maximum dose but has not been tolerating metformin, even in an extended-release formulation	• Ms. W’s NP checks in with her physician and they decide to e-consult an endocrinologist • The endocrinologist reviews the case and suggests a trial of sitagliptin	• Ms. W’s health coach calls her in 1 month; Ms. W is tolerating the sitagliptin but frequently missing doses; the health coach forwards an adherence support app for Ms. W’s smartphone • The health coach also reminds Ms. W that she is due for a retinal screening test and sends her the app for this	• Ms. W’s hemoglobin A1C in 6 months is down to 7.5, and she is no longer having recurrent yeast infections
Acute, complex care: • Ms. W develops fever and shortness of breath	• Ms. W emails the triage line for her practice, flagging the message as urgent • The triage nurse forwards the email to her physician	• Her physician calls Ms. W 30 min later, takes a detailed history over the phone, and develops a differential that includes upper respiratory infection (including influenza), pneumonia, interstitial lung disease, and pulmonary embolus • She asks Ms. W to come in later that day for an exam	• On exam, Ms. W is mildly tachypneic, with a heart rate of 112 and an oxygen saturation of 92% • She has diffuse rales on her lung exam • Her physician e-consults with a rheumatologist and pulmonologist, and they agree that a chest CT is the next step	• Ms. W’s health coach and LICSW provide emotional and logistical support to help secure a work excuse and ensure that Ms. W gets help with her children	• With virtual support from specialists, Ms. W’s physician diagnoses her with lupus, starts her on prednisone, and arranges for an in-person visit with a rheumatologist • At that visit, the rheumatologist selects appropriate pharmacotherapy with a recommended monitoring schedule • Ms. W’s primary care team supports her on this medication with occasional e-consultation with a rheumatologist

*CBT* cognitive behavioral therapy, *LICSW* licensed independent clinical social worker, *NP* nurse practitioner, *PHQ* Patient Health Questionnaire, *UTI* urinary tract infection

1. *Payment must support the primary care functions and reward value, facilitating a paradigm shift away from visit-based healthcare*. A few enlightened, integrated healthcare systems have redistributed fee-for-service revenue in ways that support greater investment in primary care. Some newer primary care organizations have achieved better financing for primary care within a fee-for-service payment model by charging patients a supplemental or “concierge” fee. Most of the high-functioning models of primary care in the U.S. that we are aware of, however—including established healthcare systems, such as Kaiser Permanente (KP) and the Southcentral Foundation (SCF) of Alaska, and newer, for-profit, direct primary care companies—are in some way paid on a capitated basis (either with global capitation or sub-capitation for primary care with or without risk sharing). Primary care investment in these systems generally amounts to about 10% of the total costs of healthcare (roughly twice the national average), and is more than offset by reductions in total medical expenditure.^34^
  With the transition away from rewarding the volume of visits, there will be a shift in patient relationships analogous to the shift in customer relationships that industries like banking have experienced over the last two decades, away from punctuated, physical, in-person interactions, towards continuous, virtual relationships. The established healthcare systems mentioned above are already well along in this journey. For example, Kaiser Permanente has predicted that the majority of its patient interactions in 2016 will be virtual.^35^ Recognizing the necessity of payment reform, the Centers for Medicare & Medicaid Services (CMS) are experimenting with, and encouraging, a form of capitation involving upfront per-member/per-month (PMPM) payments based on patient complexity, for those practices willing to provide access and continuity, care management, coordination and comprehensiveness, patient engagement, and planned care and population health.^36^
2. *Relationships will continue to serve as the bedrock of value in primary care, and will increasingly be enhanced by teams, improved clinical operations, and technology, with patients and non-physicians assuming an ever-increasing role in most aspects of healthcare*. With growing emphasis on outcomes over processes of care, provider organizations must increasingly focus on the performance of teams and the broader organization, as opposed to individual clinicians.^37^ As the day-to-day experience of primary care has witnessed a seemingly constant increase in administrative work pushed to the desks (or screens) of physicians, physician productivity has shown few of the gains seen in most industries over the last few decades.^28^ Reversing this trend will require thoughtful approaches to triage and task redistribution so that, over time, more and more routine, algorithmic care will be standardized and handled by non-physicians and technology,^38^ thereby diminishing the volume of non-value-adding work that contributes to physician burnout. Generalist physicians will continue to play an important role in the diagnosis and management of complex and/or high-acuity clinical presentations, but will cede most of the other roles in primary care that other team members, including patients, are better-suited to play.
  Decades of experience in service industries have demonstrated that customers, aided by well-designed technology and customer service professionals, can reliably take on tasks of data entry, appointment scheduling, and task follow-up. Accomplishing this shift towards a “co-production” model^39^ in healthcare will require technology tools that profoundly transcend the current generation of electronic health records, which are largely optimized for revenue capture in a fee-for-service billing paradigm. Healthcare organizations will need to adopt approaches to managing teams that enable the type of constant, rapid, data-driven improvement common in consumer internet businesses. While there will always be longitudinal records of individuals’ health information, patients will have better access to and control over these data. Meanwhile, in order to thrive in value-based payment environments while responding to increasing consumerism, healthcare organizations will capitalize on the potential created by the information technology boom by optimizing for rapid, reliable information exchange; care coordination and task management; patient triage and routing; and the use of artificial intelligence to facilitate clinical and organizational learning as well as diagnosis, monitoring, and surveillance.^40^
3. *Generalist physicians will increasingly focus on high-acuity and high-complexity presentations, and primary care teams will increasingly collaborate to manage conditions that specialists managed in the past*. There will be increasingly sophisticated approaches to segmenting and triaging the general population into different categories and levels of risk and psychosocial complexity, with different tailored approaches for each level.^41^^,^ 42 As routine and mostly algorithmic care is provided by other team members,^38^ generalist physicians will focus on “high stakes” patients such as those approaching the end of life and those with severe illness, uncertain diagnoses, and atypical clinical courses. Less expensive, non-hospital settings will be created to care for these patients, such as day treatment units or procedure units attached to primary care practices.^43^ Legitimate concerns might be raised about outpatient generalists’ preparedness for more complex caseloads after decades of adapting a “triage” mentality to survive clinic sessions with back-to-back 15-minute patient visits. We believe, however, that the reduced burden of algorithmic, routine and administrative work, the increased access to virtual “curbsides” by sub-specialists as in the e-consult model,^44^ and the reduced time pressure of a less visit-based approach to patient care will combine to afford generalists the time and enabling environment to safely and reliably handle complex diagnosis and management—and that this transition will improve satisfaction for physicians and patients alike.
  Meanwhile, better technology, payment arrangements, and clinical operations will facilitate more seamless integration and coordination between primary care teams, generalists, and specialists—after all, the primary care functions do not distinguish between types of physicians. With the push for efficiency and convenience, it is difficult to imagine that patients will continue to see different specialists for the routine prevention, diagnosis, and treatment of even relatively complex illnesses such as hepatitis C virus or atrial fibrillation. Initiatives such as Project ECHO^45^ and the UCSF^44^ experience with e-consults have demonstrated how clinicians can collaborate outside the context of physical referrals and visits to provide safe, high-quality, and convenient care to patients with complex conditions. For patients with extremely complex or technology-dependent illness such as severe chronic psychosis, short-bowel syndrome, or end-stage renal disease, it is most likely that there will be “reverse integration,” whereby the primary care functions are served in the specialized setting rather than requiring patients to separately go to a primary care setting.
4. *Primary care teams will develop an increasing ability to support health and wellness, not only by ably managing most routine mental illness, but also with increasingly refined approaches to supporting patients’ healthy lifestyles, oral health, and vision, and by more effectively integrating with other social services*. Patients have suffered from compartmentalized approaches to healthcare and social services that treat people as collections of separate medical and psychiatric conditions and social challenges, rather than taking a comprehensive, integrated, holistic approach to supporting their health and well-being. While human behavior is a complex phenomenon, influenced by the dynamic interplay between individuals’ genetics, environment, social circumstances, and physical and mental health, there is considerable room for primary care to expand its capacity to support patients in making healthy lifestyle choices, particularly with the transition to a continuous, virtual relationship.^46^ Access to evidence-based behavioral health interventions such as mindfulness and meditation, cognitive behavioral therapy for insomnia, and the Diabetes Prevention Program for weight management has been limited by reimbursement policies and service availability, but changes to CMS policies and innovative businesses may soon make them more widely available for referral or seamless integration with primary care.^47^ Meanwhile primary care teams, particularly those in integrated systems at risk for emergency room visits and hospitalizations, will bolster their capacity to administer screening and brief intervention for alcohol use disorder, pharmacotherapy for opioid use disorder, and collaborative care models for treating depression and anxiety.^48^
  Further, primary care practices, particularly those serving vulnerable, underserved patient populations, may capitalize on new technologies that are reducing the cost and space needed for specialized eye, ear, and oral health services. Problems with vision,^49^ hearing,^50^ and dentition^51^ are major contributors to chronic illness. New technologies for refraction make it possible for primary care practices to offer prescriptions for eyeglasses, while other technologies can screen for diabetic retinopathy.^52^ In some areas, primary care physicians are learning to administer dental anesthesia so they can remove diseased teeth.^53^ Pediatricians are routinely administering fluoride varnish. More of this care could occur within primary care physicians’ offices if payment were aligned with adding team members to take on these tasks.
  For patients with complex care needs from advanced mental illness and/or social challenges, there will continue to be a role for expanded primary care teams to more effectively integrate care with other social services, such as public mental health case work, housing, and employment and disability services.^54^^,^ 55

## CONCLUSION

We have focused on the justifications for the primary care revolution and how it will manifest in terms of improved care, leading to better health outcomes and patient experience at a sustainable cost. Achieving this transformation in clinician and patient activity and interaction, however, will require more than better technology and payment approaches. This change will require highly effective leadership, management, advocacy, and continuous process improvement from the front lines of care to the most senior levels of management and policymaking.^56^ As these skills and competencies have not been emphasized in the education of healthcare professionals, this transformation must be supported by considerable evolution in workforce training,^57^^,^ 58 and it is essential that trainees receive clinical training in organizations that model the future of high-value care delivery.^58^ Change is never easy, and dramatic change to something as personal as healthcare is likely to be accompanied by considerable distress for all involved, particularly for those whose livelihood is at stake. Nonetheless, we believe that physicians, particularly those early in training or practice, should view this revolution with considerable optimism and excitement, for it holds the promise not only of considerable improvement in the experience of our daily clinical work, but also of our profession drawing closer to its highest ideals of humanism and scientific rigor.
